# Sinapine Thiocyanate Ameliorates Vascular Endothelial Dysfunction in Hypertension by Inhibiting Activation of the NLRP3 Inflammasome

**DOI:** 10.3389/fphar.2020.620159

**Published:** 2021-02-09

**Authors:** Yang Liu, Hong-lin Yin, Chao Li, Feng Jiang, Shi-jun Zhang, Xin-rong Zhang, Yun-lun Li

**Affiliations:** ^1^ First Faculty of Clinical Medicine, Shandong University of Traditional Chinese Medicine, Jinan, China; ^2^ ICU, Affiliated Hospital of Shandong University of Traditional Chinese Medicine, Jinan, China; ^3^ Faculty of Traditional Chinese Medicine, Shandong University of Traditional Chinese Medicine, Jinan, China; ^4^ Experimental Center, Shandong University of Traditional Chinese Medicine, Jinan, China; ^5^ Cardiovascular Department, Affiliated Hospital of Shandong University of Traditional Chinese Medicine, Jinan, China

**Keywords:** vascular endothelial dysfunction, hypertension, NLRP3 inflammasome, inflammation, sinapine thiocyanate

## Abstract

The increase of blood pressure is accompanied by the changes in the morphology and function of vascular endothelial cells. Vascular endothelial injury and hypertension actually interact as both cause and effect. A large number of studies have proved that inflammation plays a significant role in the occurrence and development of hypertension, but the potential mechanism between inflammation and hypertensive endothelial injury is still ambiguous. The purpose of this study was to explore the association between the activation of NLRP3 inflammasome and hypertensive endothelial damage, and to demonstrate the protective effect of sinapine thiocyanate (ST) on endothelia in hypertension. The expression of NLRP3 gene was silenced by tail vein injection of adeno-associated virus (AAVs) in spontaneously hypertensive rats (SHRs), indicating that activation of NLRP3 inflammasome accelerated hypertensive endothelial injury. ST not only protected vascular endothelial function in SHRs by inhibiting the activation of NLRP3 inflammasome and the expression of related inflammatory mediators, but also improved AngII-induced huvec injury. In summary, our results show that alleviative NLRP3 inflammasome activation attenuates hypertensive endothelial damage and ST ameliorates vascular endothelial dysfunction in hypertension via inhibiting activation of the NLRP3 inflammasome.

## Introduction

Hypertension is a multifactorial cardiovascular syndrome with progressive functional or organic impairment, and it is one of the most common chronic diseases worldwide (Doyle, 1991; Elliott, 2007; Scheltens et al., 2010). Hypertension affects approximately 300 million people in China, and with increasing prevalence, this disease has become a burden on our families and society (Wang et al., 2017). Increasing research on the pathogenesis of hypertension has revealed that hyperactivity of the sympathetic nervous system and renin-angiotensin aldosterone system (RAAS), water-sodium retention caused by the kidney, insulin resistance, and cell membrane permeability changes are involved in the occurrence and development of hypertension (Zhang et al., 2019). Additionally, the vascular endothelia, which is the largest endocrine and paracrine organ in the human body with a variety of physiological functions, contributes to the process of hypertension when it becomes damaged or dysfunctional (Konukoglu and Uzun, 2017). Studies have shown that the occurrence and development of hypertension is accompanied by the involvement of inflammatory factors, among which the nucleotide-binding leucine-rich repeat receptor pyrin domain-containing-3 (NLRP3) inflammasome is intricately involved in the pathogenesis of hypertension.

The NOD-like receptor (NLR) is a pattern recognition receptor (PRR) family member, and these receptors are found in the cytosol and are able to identify pathogen-associated molecular patterns (PAMPs) and damage associated molecular patterns (DAMPS) (Adachi and Tsuda, 2019). NLRP3 is the most studied member in the NLR family, and it is expressed in granulocytes, monocytes, dendritic cells, T cells, and epithelial cells (Eren and Özören, 2019). The NLPR3 inflammasome consists of the NLRP3 receptor, the adaptor apoptosis-associated speck-like protein containing a CARD (ASC), and the effector cysteinyl aspartate specific proteinase (caspase-1). NLRP3 is a tripartite protein that includes carboxy-terminal leucine-rich repeats (LRRs), a nucleotide-binding oligomerization domain (NOD, also NACHT domain), and an amino-terminal pyrin domain (PYD) (Liu et al., 2019). Through PYD-PYD interaction, the ASC is recruited, and then its configuration change subsequently promotes the recruitment of pro-caspase-1 via CARD-CARD interaction, which results in the production of activated caspase-1 (Zheng et al., 2013), which can cleave pro-interleukin-1 beta (pro-IL-1β) and pro-interleukin-18 (pro-IL-18) to secrete mature inflammatory cytokines IL-1β and IL-18 (Satoh et al., 2014). Dalekos et al. found that the serum IL-1β level in patients with essential hypertension was significantly increased, suggesting a certain correlation between inflammatory mediator IL-1β and hypertension (Dalekos et al., 1996). It has been reported that high-salt-induced hypertension activates the sympathetic nervous system through nuclear factor-κB (NF-κB) and increased NLRP3 and IL-1β. Inhibiting NF-κB activity reduced NLRP3 and IL-1β levels, thereby reducing hypertension (Qi et al., 2016). These studies suggest that the NLRP3 inflammasome is related to the occurrence and development of hypertension. Krishnan et al. noted that MCC950, an inhibitor of the NLRP3 inflammasome, was effective at reducing blood pressure (BP) and limiting renal inflammation, fibrosis, and dysfunction in mice with established hypertension. Their study provides a proof-of-concept that the pharmacological inhibition of the NLRP3 inflammasome is a viable anti-hypertensive strategy (Krishnan et al., 2019).

The vascular endothelia is composed of single flat cells lining the surface of the vascular lumen, which regulates vascular tension and maintains vascular structural stability (Zhong et al., 2018). Endothelial dysfunction and/or structural damage may lead to increased systemic vascular resistance, which results in hypertension (Perticone et al., 2010). Derived from endothelial nitric oxide synthase (eNOS), nitric oxide (NO) released from endothelial cells causes smooth muscle relaxation and subsequent vasodilation (ALrefai et al., 2016; Santos-Parker et al., 2017). Inhibition of NO is associated with hypertension, and this imbalance between vasoconstriction and vasodilation caused by endothelial damage is one of the important pathophysiological mechanisms of hypertension (Schlaich et al., 2004). Inflammatory mediators and inflammasomes alter the rate of synthesis and degradation of vasoconstrictors and vasodilators, especially NO, which regulates the vascular endothelial function and changes the BP. Jiabao Li et al. demonstrated that the application of exogenous sodium hydrosulfide (NaHS) can significantly reduce the systolic blood pressure and improve the function of damaged vascular endothelia. Hydrogen sulfide (H_2_S) repaired damaged vascular endothelia and significantly reduced systolic blood pressure by suppressing the activation of the NLRP3 inflammasome in spontaneously hypertensive rats (SHRs). In human umbilical vein endothelial cells (HUVECs), H_2_S also significantly ameliorated angiotensin II (AngII)-induced endothelial injury by reducing the NLRP3 inflammasome activity (Li et al., 2019a). A promising treatment for hypertension involves inhibiting the activity of the NLRP3 inflammasome and stopping the vicious cycle of inflammation and endothelial injury.

Sinapine is a quaternary amine alkaloid that is widely found in cruciferous plants and has a broad range of pharmacological effects (Guo et al., 2016). Sinapine often combines with glycosides, organic acids, esters, amides, and thiocyanates (Bhinu et al., 2009). It was reported that *Uncaria rhynchophylla* total alkaloids and *Semen Raphani* soluble alkaloid were combined and used to treat the vascular endothelial cells in N′-nitro-l-arginine-induced hypertensive rats, and it satisfactorily reduced blood pressure with endothelial protection. Sinapine thiocyanate (ST) is the main active component of sinapine in *Semen Raphani*. Studies have confirmed that ST has an exact effect on lowering blood pressure, and its mechanism is related to inhibiting the secretion of adhesion factors by vascular endothelial cells and suppressing inflammation (Li et al., 2015). ST was able to downregulate the expression of coagulation-related factors in dysfunctional vascular endothelial cells, thereby suppressing the prothrombotic state caused by inflammatory injury to the vascular endothelia (Li et al., 2017). In addition, previous studies have revealed that sinapine is a natural antioxidant that can scavenge free radicals and has anti-aging (Qun and Ruiqi, 1999) and anti-inflammation effects (Zhang and Shen, 1996).

The current study focused on the relationship between the NLRP3 inflammasome and hypertensive endothelial dysfunction, and explored whether the natural drug ST could normalize the function of the vascular endothelia by reducing NLRP3 inflammasome activation. Through inhibiting the activation of the NLRP3 inflammasome, ST may be a promising natural medicine for protection against hypertensive organ damage.

## Materials and Methods

### Cell Culture and Intervention

HUVECs were isolated from the vein of a normal human umbilical cord and cultured in endothelial cell medium (ECM) (ScienCell, USA). The cells were seeded and grown in cell culture flasks at concentrations of 7 × 10^4^ cells/ml under humidified 5% CO_2_ conditions. For initial experiments, the HUVECs were randomly divided into different groups, and AngII (5 × 10^−6^ mol/L) was used to simulate the damage of vascular endothelial cells in patients with hypertension. The 3-(4,5-dimethylazol-2-yl)-2,5-diphenyltetrazolium bromide (MTT) assay was used to obtain the optimal intervention concentration of ST, and the intervention concentration of ST was 50 mg/L.

### Animals

Forty male (females were excluded because of the effect of the menstrual cycle on blood pressure), eight-week-old SHRs were provided by Beijing Vital River Laboratory Animal Technology Co., Ltd. (Animal Qualification License No. SCXK (Beijing) 2016–0006). After the animals arrived, the outer surfaces of the boxes were disinfected by ultraviolet light, and then, five rats were placed into each cage. SHRs were divided into different groups that each contained eight rats: model group (SHR group): received an equal amount of distilled water by gavage and tail vein injection every day; drug intervention group (SHR + ST group): each rat was treated with 8.54 mg/kgd ST by gavage for eight weeks; SHR + NLRP3-adeno-associated virus (AAV) group (SHR + NLRP3-AAV group): each rat received a single tail vein injection of NLRP3-AAV (approximately 1 × 10^11^ transducing units in 1 ml of saline solution); SHR + Control-AAV group: each rat received a single tail vein injection of control-AAV (approximately 1 × 10^11^ transducing units in 1 ml of saline solution). Sixteen male, 8-week-old WKY rats (Beijing Vital River Laboratory Animal Technology Co., Ltd.) were selected and used as the control group (WKY group). These rats received an equal amount of distilled water by gavage and tail vein injection every day. The tail artery systolic blood pressure (SBP) and diastolic blood pressure (DBP) (unit: mmHg) of rats were measured regularly every week during the period of mold making and during the period of drug delivery respectively. Before measuring blood pressure, the rats were placed on the operating table and heated for 10 min, to increase body temperature and vasodilation. A tail-cuff sphygmomanometer with an automated system photoelectric sensor (ALC-Non-Invasive Blood Pressure System, Shanghai Alcott Biotech Co., Ltd., Shanghai, China) was used to measure the blood pressure, and the action should be gentle in order to avoid rats irritation. All rats were measured 3 times in parallel, and data was collected as a mean.

### Enzyme-Linked Immunosorbent Assay (ELISA)

The levels of mature-IL-1β, mature-IL-18 and ET-1 protein in the rat serum or culture supernatant were determined using commercially available ELISA IL-1β, IL-18 and ET-1 kits (Elabscience, Wuhan, China) according to the manufacturer’s protocols. IL-1β, IL-18 and ET-1 levels were determined by comparing the samples to the standard curve generated by the kit.

### NO Assay

NO levels in the culture supernatant and rat serum were determined using an NO detection kit (Nanjing Jiancheng Bioengineering Institute) according to the instructions. The samples and reagents 1 (R1) and R2 were mixed, and then incubated for 60 min at 37 °C. R3 and R4 were then added, the solution was mixed for 30 s, incubated at room temperature for 40 min, and then centrifuged for 10 min at 3,500 rpm. Next, 0.5 ml supernatant was removed, mixed with a chromogenic agent, and incubated for 10 min at room temperature. The colorimetric result was obtained at a wavelength of 550 nm and optical diameter of 0.5 cm. The optical density was measured, and the NO content was calculated.

### Protein Extraction and Western Blot

Both the levels of NLRP3, pro-caspase-1, eNOS (total), eNOS (phospho Ser1177) (p-eNOS) and NF-κB (p65) in rats and NLRP3, pro-caspase-1, eNOS, p-eNOS, NF-κB (p65) and tumor necrosis factor-α (TNF-α) in cells were detected by western blot. Tissue homogenates were prepared in 1 nM phenylmethanesulfonyl fluoride (PMSF) lysis buffer (Beyotime, Shanghai, China). The collected cell pellet was also lyzed with PMSF to extract the protein. The protein concentrations in the cell lysates and tissue homogenate were determined with a bicinchoninic acid (BCA) kit (Beyotime, Shanghai, China). Proteins were denatured, and equal amounts of proteins were electrophoresed in 8% or 10% bis-Tris/polyacrylamide gels (Beyotime, Shanghai, China) and then transferred to polyvinylidene fluoride (PVDF) membranes (Millipore Co., Ltd.). The membranes were blocked for 1 h in blocking solution (TBST containing 5% skim milk powder and 0.1% Tween-20) and incubated overnight at 4 °C with anti-NLRP3, anti-caspase-1 antibody (Abcam, Cambridge, United Kingdom) and anti-NF-κB (p65), anti-TNF-α antibody (CST, United Kingdom), anti-eNOS (phospho Ser1177) antibody (GeneTex, United States) at 1:1,000 and anti-eNOS antibody (CST, United States) at 1:2000 in primary antibody diluent (Meilunbio, China). After that, incubation with horseradish peroxidase-conjugated secondary antibody (1:10,000 dilution in TBST containing 0.1% Tween-20) was performed at room temperature for 1 h, and immunoreactivity was detected by using enhanced chemiluminescence (Millipore Co., Ltd.). Blots were scanned and analyzed for measurement of the band intensities using UN-SCAN-IT version 5.1 software. Band intensity was calculated as follows: band intensity = sum of all pixel values in the segment selected–background pixel value in that segment.

### RNA Extraction and Quantitative Real-Time PCR Analysis

The mRNA levels of NLRP3 and caspase-1 were analyzed by RT-PCR. According to the instructions, total RNA was isolated from HUVECs and rat tissue using an RNA extraction kit (Omega, United States). This procedure was performed under RNase-free conditions. The total RNA (15 µg) was reverse transcribed to cDNA using a PrimeScript™ RT reagent kit with gDNA Eraser (TaKaRa, Kusatsu, Japan) according to the instruction manual. The specific transcripts were quantified by quantitative RT-PCR using SYBR^®^ PreMix Ex Taq™ II (TliRNaseH Plus) and analyzed with the LightCycler 480 system. Gene-specific primers were synthesized by SparkJade (Shandong, China), and the forward (F) and reverse (R) primers are listed in Table 1. The mRNA levels were normalized to the β-actin mRNA level.

**TABLE 1:** Primer sequences.

Subjects	Genes	Primers	Primer sequences (5′-3′)
SHRs	β-actin	β-actin-F	CCC​ATC​TAT​GAG​GGT​TAC​GC
		β-actin-R	TTT​AAT​GTC​ACG​CAC​GAT​TTC
	NLRP3	NLRP3-F	GCA​GCG​ATC​AAC​AGG​CGA​GAC
		NLRP3-R	TCC​CAG​CAA​ACC​TAT​CCA​CTC​CTC
	caspase-1	caspase-1-F	CTG​GAG​CTT​CAG​TCA​GGT​CC
		caspase-1-R	CTT​GAG​GGA​ACC​ACT​CGG​TC
HUVECs	GAPDH	GAPDH-F	AGA​AGG​CTG​GGG​CTC​ATT​TG
		GAPDH-R	AGG​GGC​CAT​CCA​CAG​TCT​TC
	NLRP3	NLRP3-F	GTT​GTG​TGA​AAC​GCT​CCA​GCA​T
		NLRP3-R	TGC​TTC​AGT​CCC​ACA​CAC​AG
	caspase-1	caspase-1-F	TCC​GTT​ATT​CCG​AAA​GGG​GC
		caspase-1-R	TGA​GGA​TGT​GGG​CAT​AGC​TG

### Migration Assay

The cell migration rate was determined using a transwell chamber (Corning, USA). After HUVECs were diluted to 1 × 10^5^/ml with ECM, 0.5 ml ECM was added to the lower compartment, and 0.2 ml cell suspension was added to the upper compartment. After incubating for 6 h at 37 °C, the culture medium was removed by aspiration, and the remaining cells on the filter membrane of the upper side were gently wiped with a dry cotton swab. The cells were fixed with 4% paraformaldehyde for 30 min and stained with hematoxylin and eosin for 30 and 10 min, respectively. The number of HUVECs that migrated from the upper compartment to the lower compartment was counted.

### Adhesion Assay

Fibronectin was diluted with Dulbecco’s modified Eagle’s medium (DMEM)·F12 (at a ratio of 1:9), and tiled in a 24-well plate for 12 h at 4 °C. HUVECs that underwent different treatments were inoculated on the 24-well plate, and then cultivated for 1 h at 37 °C. Unattached HUVECs were removed with phosphate-buffered saline rinses. The cells were fixed with 4% paraformaldehyde for 10 min, and then stained with hematoxylin staining solution and eosin staining solution for 30 and 10 min, respectively. The number of adherent cells was counted under the microscope.

### *In vitro* Tube Formation Assay

Network formation capacity was detected using a tube formation assay kit (Sigma-Aldrich, USA). First, 0.05 ml basement membrane extract (BME)/per well was added to a 96-well plate, which was then incubated for 1 h at 37 °C. HUVECs were diluted to 1 × 10^5^/ml with ECM, and 0.1 ml cell suspension was added to the 96-well plate. After 6 h, the number of tubular structures was counted.

### Preparation of Thoracic Aortic Rings and Detection of Tension in Isolated Thoracic Aortic Rings

The thoracic aorta was removed and transferred to a Petri dish filled with cold oxygenated modified Krebs solution. Then, the surrounding fat and connective tissue were removed from the aorta under a dissecting microscope, and the isolated aorta was transversely cut into 4 mm-length rings. The prepared aorta rings were horizontally suspended on a tension transducer so that one end of the aorta ring was located on the fixed hook and the other end was connected with the tension transducer. The aorta rings were immersed into organ bath chambers filled with modified Krebs solution. The solution was continuously gassed with 95% O2/5% CO2 and maintained at 37 °C. The tension change was recorded by the LabChart multi-channel signal analysis system. The resting tension was adjusted to 1 g. Arteries were exposed to 10^–6^ mol/L phenylephrine (Phe) to induce contraction. After the tension baseline became stable, the maximum contractile force D0 was recorded, and then, 10^−9^–10^–5^ mol/L acetylcholine (Ach) was administered. The changes in vasodilation under cumulative concentration were observed, and the maximum vasodilation intensity D1 of each concentration gradient was recorded.

The rings were rinsed 3 times, for 10 min each time with fresh Krebs reagent. Then, the tension of the vascular ring was balanced to the initial state again, and 10^–6^ mol/L Phe reagent was provided again to stimulate vasoconstriction. After the contractile tension was stable, the maximum contraction value D2 was recorded. Then, 10^−9^–10^–5^ mol/L sodium nitroprusside (SNP) was intermittently administered. The vasodilation activity was observed under the cumulative concentration. After stabilization, the maximum relaxation value D3 of each concentration was recorded. Aortic vasodilation capacity is expressed as a percentage of vasodilation tension in contraction induced by Phe stimulation, that is, Ach relaxation is calculated as (D0-D1)/D0 relaxation * 100%, and SNP relaxation is calculated as (D2-D3)/D2 relaxation * 100%.

### HE Staining

After deparaffinization and rehydration, 5 μm longitudinal sections were stained with hematoxylin solution for 5 min and then dipped in 1% acid ethanol (1% HCl in 70% ethanol) 5 times. After that, the sections were rinsed in distilled water. Then, the sections were stained with eosin solution for 3 min, followed by dehydration with graded alcohol and clearing in xylene. The mounted slides were then examined and photographed using a fluorescence microscope. The staining intensity was analyzed by Image-Pro Plus 6.0 software and expressed as the iod value.

### Masson Trichrome Staining

Masson trichrome staining was conducted using a ready-to-use kit (Trichrome Stain (Masson) Kit, HT15, Sigma-Aldrich). Briefly, tissue sections (5-μm thickness) were cut and placed on standard microscopy slides. After deparaffinisation and rehydration, the slides were immersed in Bouin’s solution (HT 10132, Sigma-Aldrich) at 56 °C for 15 min. Then, the slides were washed with tap water for 5 min. The sections were stained in Weigert’s hematoxylin for 5 min, and then washed again with tap water for 5 min and rinsed in distilled water. The slides were stained in Biebrich scarlet-acid fuchsin for 5 min, rinsed in distilled water, incubated in phosphotungstic-phosphomolybdic acid for 5 min, dyed with aniline blue for 5 min, and fixed in 1% acetic acid for 2 min. Finally, the slides were rinsed in distilled water, dehydrated, and mounted.

### Statistical Analysis

The data are expressed as the mean ± SD of triplicate experiments and were analyzed with SPSS version 17.0 software (SPSS Inc., Chicago, IL, United States). Statistically significant values were determined using analysis of variance (ANOVA) and Dunnett’s post hoc test, and *p*-values of less than 0.05 were considered statistically significant.

## Results

### The Activation of the NLRP3 Inflammasome Played a Crucial Role in Endothelial Dysfunction in SHRs

**FIGURE 1:**
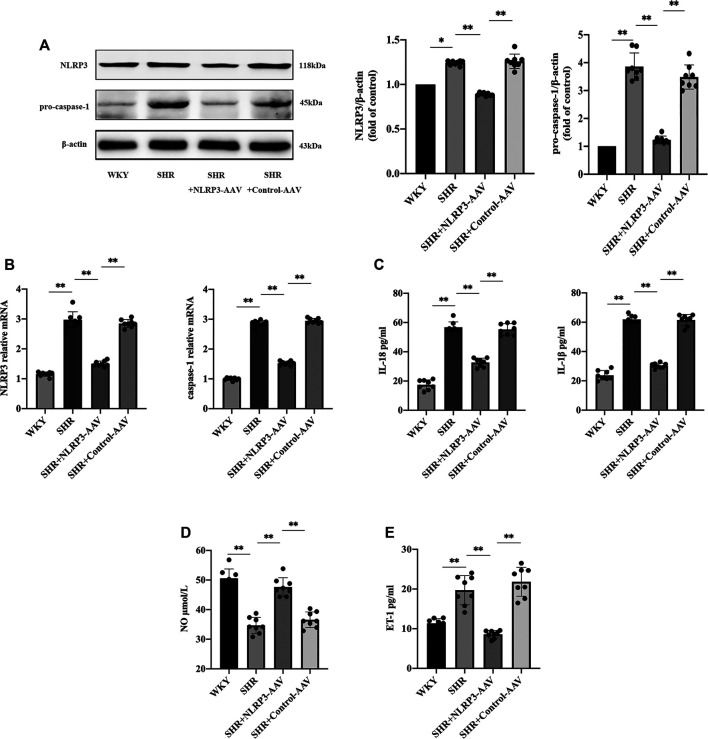
The activation of NLRP3 inflammasome played a crucial role in endothelial dysfunction in SHRs (*n* = 8). **(A)** Western blot analysis of the expression of NLRP3 and pro-caspase-1 in WKY, SHR, SHR + NLRP3-AAV and SHR + Control-AAV groups. The data were presented in fold of control. **(B)** PCR analysis of the expression of NLRP3 and caspase-1 in above four groups. **(C)** ELISA analysis of the levels of IL-1β and IL-18 in above four groups. **(D)** ELISA analysis of the level of NO in above four groups. **(E)** ELISA analysis of the level of ET-1 in above four groups. All data were shown as mean ± standard deviation (SD) (error bars) from eight independent biological repeats experiments, each of which included three technical repeats. ^*^ *p* < 0.05; ^**^ *p* < 0.01.

Endothelial dysfunction contributes to hypertension, and previous studies have found that elevated blood pressure was accompanied by increased levels of the NLRP3 inflammasome (Wang et al., 2018a). Therefore, we questioned whether there was a link between the NLRP3 inflammasome and vascular endothelial damage in hypertension. On this basis, we first studied the changes in the vascular endothelia during hypertension. The results of the vascular endothelial function test showed that the level of NO (Figure 1D) was decreased in SHRs, but the level of endothelin-1 (ET-1) (Figure 1E) was increased. Evidently, there was an imbalance between vasoconstrictors and vasodilators released by the endothelia, which indicated that hypertension is characterized by excessive vasoconstriction induced by vascular endothelial dysfunction.

We then observed changes in the NLRP3 inflammasome in SHRs. Western blot and PCR results showed a boost in NLRP3 and caspase-1 (Figures 1A,B) at the same time that endothelial injury occurred, and ELISA showed increased levels of inflammatory factors IL-1β and IL-18 (Figure 1C), which were induced by the NLRP3 inflammasome. In addition, in order to confirm the causal association between NLRP3 inflammasome activation and endothelial dysfunction, we injected NLRP3-AAV to inhibit the expression of NLRP3. Measurement of the level of NO indicated that it increased (Figure 1D), and ET-1 (Figure 1E) decreased, suggesting that activation of the NLRP3 inflammasome could result in vascular endothelial damage. The above results suggested that the NLRP3 inflammasome can be targeted to reduce vascular endothelial injury in hypertension.

### ST Improved the Endothelial Function in SHRs

**FIGURE 2:**
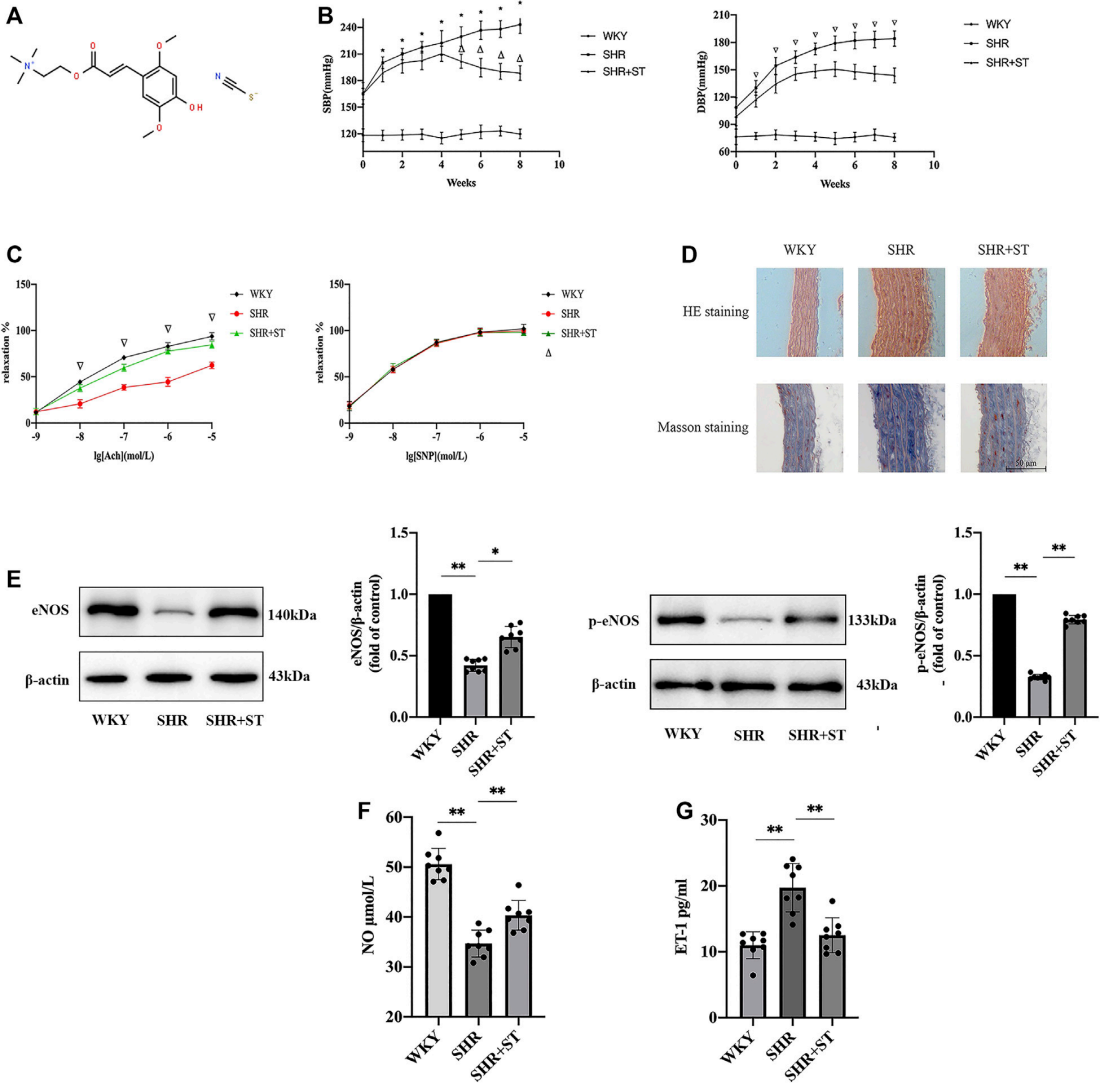
ST improved endothelial function in SHRs (*n* = 8). **(A)** The chemical structure of ST. **(B)** Effect of ST treatment on blood pressure during eight weeks. ^*^ *p* < 0.05, SHR group compared with WKY group at SBP; ^△^ *p* < 0.05, SHR + ST group compared with SHR group at SBP; ^∇^ *p* < 0.05, SHR group compared with WKY group and SHR + ST group compared with SHR group at DBP. **(C)** Detection of tension results of isolated thoracic aortic rings in above three groups. ^∇^ *p* < 0.05, SHR group compared with WKY group and SHR + ST group compared with SHR group; ^△^ *p* > 0.05, among above three groups in every concentration of SNP. **(D)** Histopathological changes of aorta were observed by light microscopy after HE staining (200×) and Masson staining (200×). **(E)** Western blot analysis of the expression of eNOS and phospho-eNOS (p-eNOS) in WKY, SHR and SHR + ST groups. The data were presented in fold of control. **(F)** ELISA analysis of the level of NO in above three groups. **(G)** ELISA analysis of the level of ET-1 in above three groups. All data were shown as mean ± standard deviation (SD) (error bars) from eight independent biological repeats experiments, each of which included three technical repeats. ^*^ *p* < 0.05; ^**^ *p <* 0.01.

The formula of ST is [C_16_H_24_NO_5_]^+^(SCN)^-^, and its chemical structure is shown in Figure 2A (Li et al., 2019b). The most important sign of hypertension is elevation of blood pressure, including SBP and DBP, mainly due to the damage of endothelial cells and continuous constriction of blood vessels (Figure 2B). The vascular endothelia maintain constant vascular tension by synthesizing and secreting vasoconstrictors and vasodilators. Therefore, we investigated the effect of ST on vascular endothelial function. Compared with the WKY group, the SHRs exhibited a significant decrease in endothelia-dependent vasodilation activity induced by Ach stimulation. After eight weeks of ST treatment, compared with the SHR group, the SHRs with ST intervention experienced restoration of sensitivity to Ach stimulation, and the endothelia-dependent vasodilation function was significantly improved.

During the non-endothelia-dependent vasodilation function trial, it was found that the aorta of the rats in each group responded well to the vasodilation activity generated by SNP stimulation at various concentrations, and the vasodilation function level of rats in each group was the same to some extent. The results indicated that the non-endothelial-dependent vasodilation function did not significantly change under the condition of hypertension, and the regulating effect of ST on the non-endothelial-dependent vasodilation function was not obvious (Figure 2C).

The HE staining indicated that the media of the aorta in the SHRs was distinctly thickened as compared with that of the WKY rats. However, medial thickening of the aorta in the SHRs was significantly ameliorated by administration of ST. In addition, Masson staining showed a large number of collagen fibers proliferating in the vascular wall of the SHRs, which proved that the occurrence of hypertension was accompanied by obvious vascular remodeling, while the intervention of ST reduced the amount of collagen fibers, and the vascular wall began to revert to a normal structure (Figure 2D). The results of western blot, NO assay, and ELISA showed that the levels of eNOS and p-eNOS both of which reduced evidently in SHRs (Figure 2E) as well as NO levels (Figure 2F) increased, and ET-1 level (Figure 2G) decreased in SHRs with ST intervention. These results suggested that ST improved vascular endothelial function in hypertension, which confirmed again the feasibility of protecting the vascular endothelial cells against damage from hypertension.

### ST Inhibited the Activation of the NLRP3 Inflammasome and Attenuated Inflammation in SHRs

**FIGURE 3:**
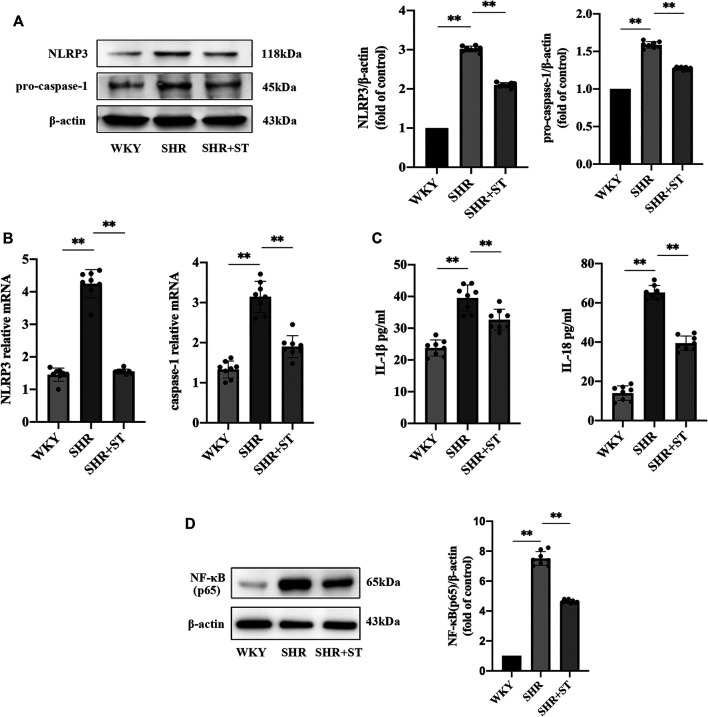
ST inhibited the activation of NLRP3 inflammasome and attenuated inflammation in SHRs (*n* = 8). **(A)** Western blot analysis of NLRP3 and pro-caspase-1 in WKY, SHR, SHR + ST groups. The data were presented in fold of control. **(B)** PCR analysis of the expression of NLRP3 and caspase-1 in above three groups. **(C)** ELISA analysis of the levels of IL-1β and IL-18 in above three groups. **(D)** Western blot analysis of NF-κB (p65) in above three groups. The data were presented in fold of control. All data were shown as mean ± standard deviation (SD) (error bars) from eight independent biological repeats experiments, each of which included three technical repeats. ^*^ *p* < 0.05; ^**^ *p* < 0.01.

Our previous studies confirmed the protective effect of ST on vascular endothelial cells in SHRs. Therefore, in order to further study the effect of ST on the NLRP3 inflammasome, we administered ST to SHRs and observed the subsequent process. The results showed that ST alleviated the inflammation in hypertension, which manifested as reduced NLRP3 and caspase-1 levels (Figures 3A,B), and demonstrated that ST had an inhibitory effect on the activation of the NLRP3 inflammasome. In addition, the ELISA results showed a decrease in IL-1β and IL-18 levels (Figure 3C). As shown in Figure 3D, the levels of NF-κB (p65) decreased after ST intervention, suggesting that the NLRP3 inflammasome-induced inflammatory pathway was the effective target of ST. These data and images suggested that although NLRP3 inflammasome activity and inflammation were hyperactive in the SHRs, ST alleviated inflammatory damage in hypertension by inhibiting the activation of the NLRP3 inflammasome.

### ST Attenuated AngII-Induced HUVECs Dysfunction

**FIGURE 4:**
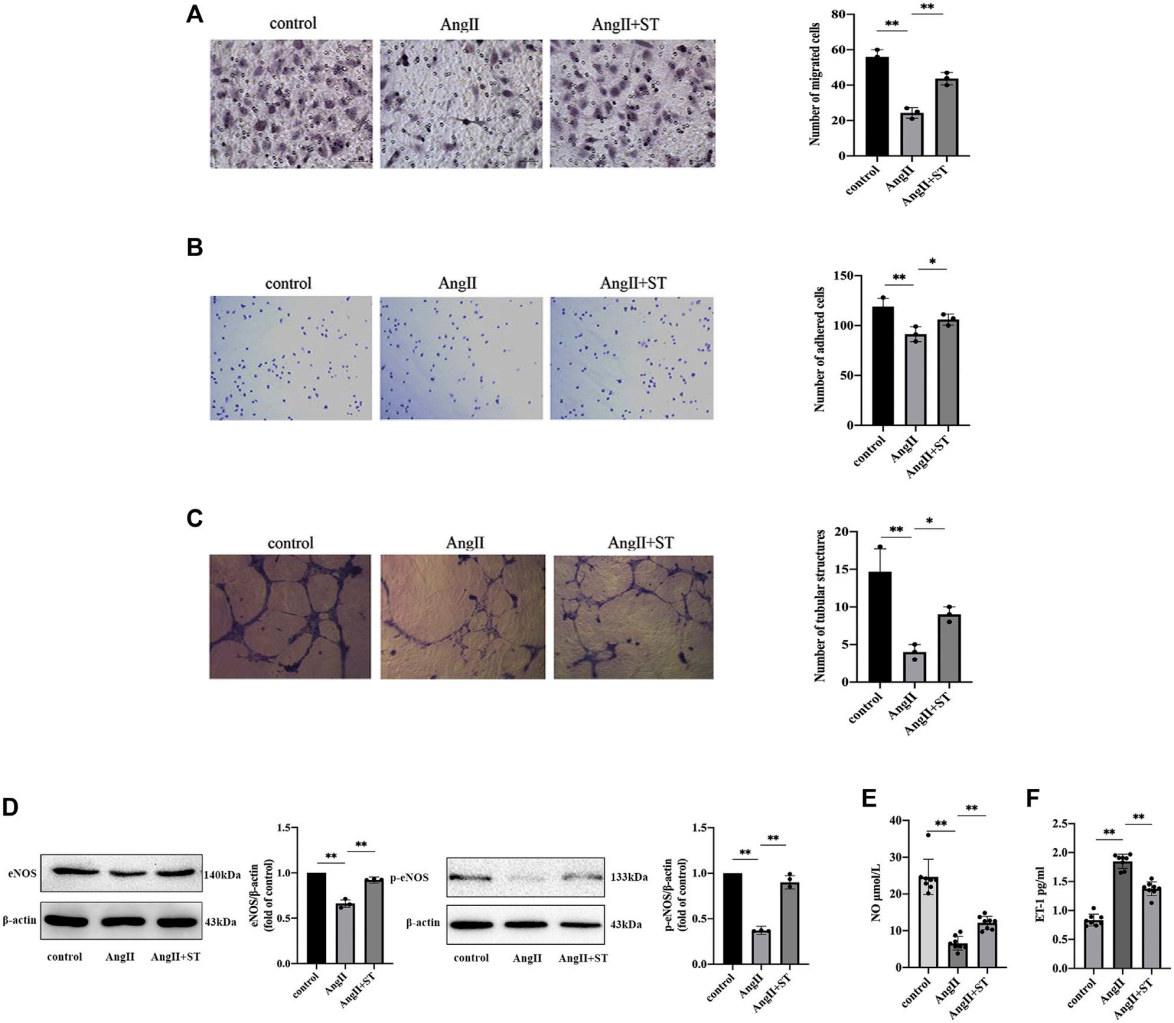
ST attenuated AngⅡ-induced HUVECs dysfunction. **(A)** Representative images and quantitative analysis of migration assay (HE stain, 100×). **(B)** Representative images and quantitative analysis of adhesion assay (HE stain, 100×). **(C)** Representative images and quantitative analysis of tube formation assay *in vitro* (200×). The number of tubular structures in the field represented the angiogenesis capacity of vascular endothelia. **(D)** Western blot analysis of eNOS and p-eNOS expression in control, AngⅡ and AngⅡ+ST groups. The data were presented in fold of control. **(E)** ELISA analysis of the level of NO in above three groups. **(F)** ELISA analysis of the level of ET-1 in above three groups. All data except ELISA analysis were shown as mean ± SD (error bars) from three independent biological repeats experiments, each of which included three technical repeats. ^*^ *p* < 0.05; ^**^ *p* < 0.01.

Endothelial function is closely related to the proliferation, migration, adhesion, and angiogenesis of vascular endothelial cells, and therefore, we investigated the effects of ST on these functions. The results showed that the number of migrating cells (Figure 4A), adhesion cells (Figure 4B), and tubular structure (Figure 4C) all decreased in AngII-induced HUVECs. However, ST increased DNA replication activity, increased migration and adhesion, and promoted the formation of tubular structures. Additionally, the levels of eNOS, p-eNOS (Figure 4D) and NO (Figure 4E) decreased, and ET-1 levels (Figure 4F) were elevated in AngII-induced HUVECs. After ST intervention, however, the levels of eNOS, p-eNOS and NO were ameliorated evidently as well as decreased ET-1, which is consistent with the results of previous experiments with animals. These results suggested that ST restored the function in vascular endothelial cells damaged by AngII.

### ST Inhibited AngII-Induced NLRP3 Inflammasome Activation in HUVECs

**FIGURE 5:**
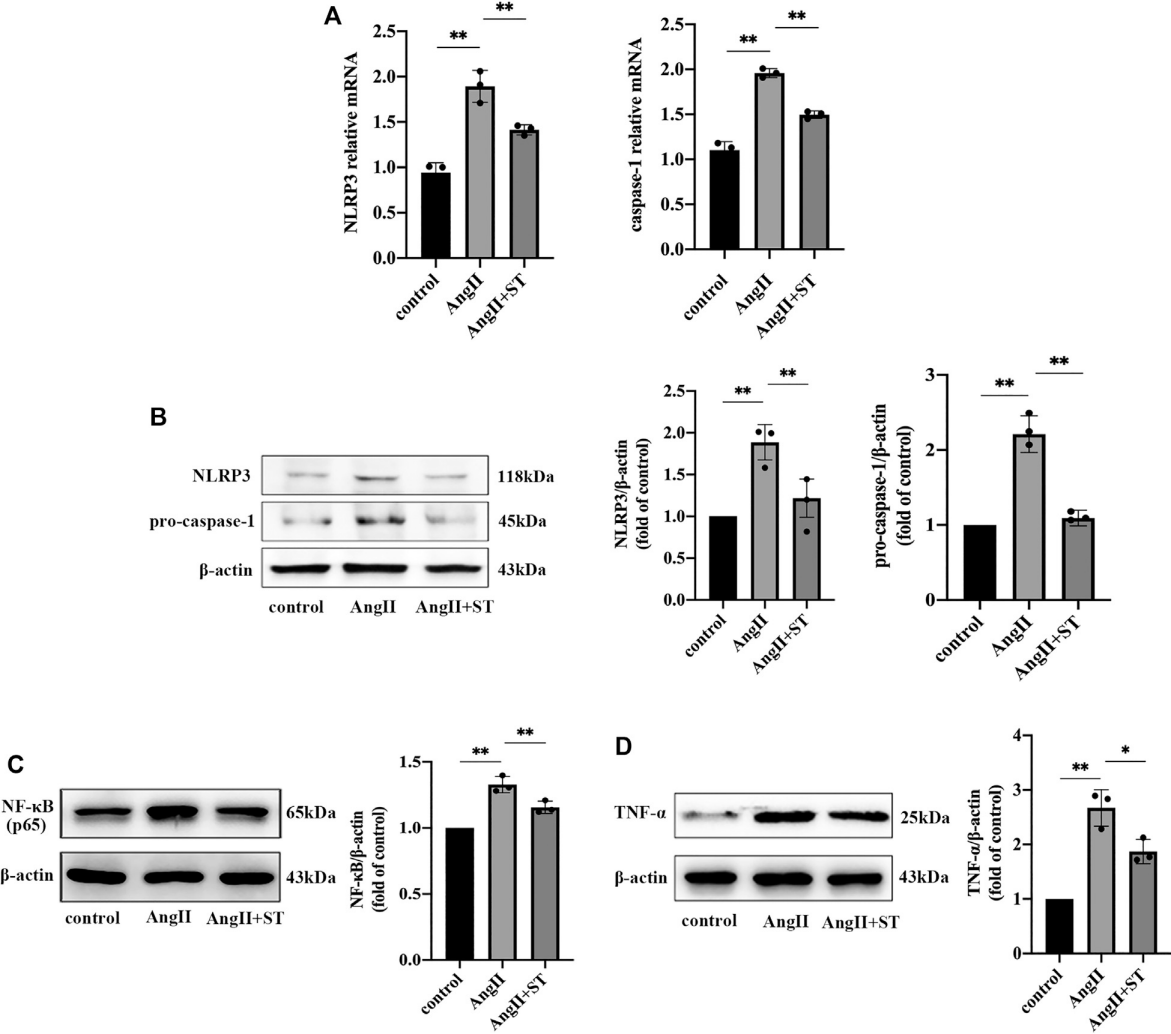
ST inhibited AngII-induced NLRP3 inflammasome activation in HUVECs. **(A)** PCR analysis of the expression of NLRP3 and caspase-1 in control, AngⅡ and AngⅡ+ST groups. **(B)** Western blot analysis of NLRP3 and pro-caspase-1 in above three groups. The data were presented in fold of control. **(C)** Western blot analysis of NF-κB (p65) in above three groups. The data were presented in fold of control. **(D)** Western blot analysis of TNF-α in above three groups. The data were presented in fold of control. All data were shown as mean ± SD (error bars) from three independent biological repeats experiments, each of which included three technical repeats. ^*^ *p* < 0.05; ^**^ *p* < 0.01.

We confirmed the protective effect of ST on AngII-induced vascular endothelial dysfunction. In consideration of the previous experiment, we suspected that the protective effect of ST on endothelia was related to fact that the activation of the NLRP3 inflammasome was restrained, and therefore, we added ST to vascular endothelial cells induced by AngII. The results showed that ST definitely suppressed activation of the NLRP3 inflammasome in the process of AngII-induced hypertensive damage. Western blot and PCR results suggested that AngII increased NLRP3 and caspase-1 levels, but after the intervention of ST, both levels dropped, which effectively illustrated that ST had an inhibitory effect on the activation of the NLRP3 inflammasome (Figures 5A,B). In addition, levels of NF-κB (p65) and TNF-α both rose in AngII-induced HUVECs, but subsequently decreased after ST intervention (Figures 5C,D), which strongly verified once more that the NLRP3 inflammasome is the pharmacological target of ST. These results suggested that AngII mediated NLRP3 inflammasome activity and inflammation, but ST was capable of inhibiting AngII-induced NLRP3 inflammasome activation.

## Discussion

Basic and clinical studies have proved that hypertension is a heterogeneous disease with multiple factors, links, stages, and differences between individuals (Asgary et al., 2016). Vascular endothelial dysfunction is not only the cause but the consequence of hypertension, which is an important mechanism of hypertension (Sueta et al., 2017). This study focused on the association between the NLRP3 inflammasome and hypertensive vascular endothelial injury, revealing the fact that the NLRP3 inflammasome greatly contributed to hypertensive endothelial damage. In addition, research showed that due to the anti-inflammatory effects of ST on the activation of the NLRP3 inflammasome, not only did ST reduce blood pressure and decrease the damage to endothelial cells in the SHRs, but it also ameliorated AngII-induced huvec damage. This study proved that ST has evident anti-inflammatory and protective effects on the endothelial function in hypertension, and that the NLRP3 inflammasome is also a pharmacological target of ST that can be used for effective hypertension treatment.

The vascular endothelia lines the inner surface of the vascular lumen and pervades the vascular system throughout the body. It is not only a semi-permeable barrier, but also a homeostatic organ that maintains its structure and regulates vascular tension. The vascular endothelia has a complex enzyme system and is an important metabolic and endocrine organ of the body. It can synthesize and secrete a variety of vasoactive substances, maintain vascular tension, participate in inflammation, adjust vascular growth, and regulate platelet function (Hobson et al., 2018). Under physiological conditions, the vasoactive substances released by the vascular endothelia locally maintain a certain concentration ratio to achieve a balance between vasoconstriction and vasodilation. Disruption of the balance between the two systems is an important pathophysiological mechanism for the occurrence of hypertension (He et al., 2014). Various cardiovascular system risk factors can change the function and structure of endothelial cells, resulting in loss of normal endothelial function, known as endothelial dysfunction, which is mainly characterized by repressed activation of eNOS and reduced production of NO. If the vascular tension regulation is disordered, the endothelia-dependent vasodilation function is decreased, and vasoconstriction function is increased (Widlansky et al., 2003). When the vascular wall structure microscopically changes, the organ system structure and function are damaged. The increasingly elevated blood pressure is superficial, but human health is severely threatened (Kho et al., 2018). Endothelial dysfunction is the mutual cause and effect of hypertension, which can further damage the vascular endothelia structure and aggravate endothelial dysfunction. This cyclical process greatly increases the difficulty in successfully treating hypertension.

Some researchers have proposed that hypertension is essentially the existence of chronic low-grade inflammation, and this provides new direction for the development of agents that can be used for the treatment of hypertension (David et al., 2009). Vascular endothelial injury is the result of multi-molecule and multi-pathway interaction, and studies have found that such injury is often accompanied by the involvement of various inflammatory mediators, among which the NLRP3 inflammasome is essential. Avolio et al. compared the expression of the NLRP3 inflammasome in the blood pressure-regulating regions of SHR and Wistar rats, and found that mRNA levels of NLRP3, caspase-1, and IL-1β in the amygdala, hypothalamus, and brainstem of SHR were significantly increased (Avolio et al., 2018). Qi found that the IL-1β expression in the paraventricular nucleus of high-salt diet rats significantly increased compared with normal-salt diet rats. At the same time, the mean arterial pressure, heart rate, and serum norepinephrine levels in high-salt diet rats were also remarkably elevated. In addition, when gevokizumab (IL-1β inhibitor) was injected into the paraventricular nucleus of high-salt rats, the mean arterial pressure, heart rate, and norepinephrine in the serum were successfully decreased. Furthermore, Qi investigated the effect of inhibiting NF-κB in the activation pathway of the NLRP3 inflammasome on RAAS in salt-sensitive hypertensive rats, and then found that NF-κB inhibitor significantly reduced the mean arterial pressure and norepinephrine in salt-sensitive hypertensive rats, producing similar effects as those obtained by the inhibition of IL-1β (Qi et al., 2016).

The above series of studies have shown that the occurrence of hypertension is closely related to inflammation. In the state of hypertension, there is excessive activation of the NLRP3 inflammasome in the cardiovascular regulatory center, while intervention in the activation of the NLRP3 inflammasome in the cardiovascular center to reduce the downstream inflammatory mediators and halt the inflammation damage can effectively alleviate the severity of hypertension (Shirasuna et al., 2015). In this study, the aortic vascular endothelia of Wistar rats and SHRs was selected for trial, and the results showed that the levels of NLRP3 and caspase-1 in SHRs were significantly increased, and when the NLRP3 inflammasome was clearly activated in hypertension, the downstream inflammatory factors such as IL-1β and IL-18 were also augmented. At the same time, the endothelial function of the SHRs was inhibited, with decreased endothelia-derived vasodilation factors such as eNOS and NO and increased endothelia-derived vasoconstriction factors such as ET-1.

Based on the correlation between the NLRP3 inflammasome and hypertensive endothelial dysfunction, we injected the SHRs with AAVs to inhibit the assembly of the NLRP3 inflammasome. This offset the effect of the NLRP3 inflammasome, and we found that the vasodilation factors in the SHRs were higher. It was shown that the activation of the NLRP3 inflammasome accelerated endothelial injury in hypertension and promoted an increase in blood pressure. Our data reveal the vital role of the NLRP3 inflammasome in vascular endothelial injury in SHRs, and also provide a new direction for a follow-up study on the antihypertensive effect of ST.

Previous studies proved that ST has a satisfactory antihypertensive effect (Huang et al., 2011; Boulghobra et al., 2020), and we also found that ST reduced inflammatory injury to a certain extent (Li et al., 2017). We chose the aorta from WKY rats and SHRs to extract vascular endothelial cells for subsequent experiments, and after ST intervention in SHRs, the expression of NLRP3 and caspase-1 decreased with the suppression of NLRP3 inflammasome activity. Additionally, inflammatory factors such as NF-κB (p65), TNF-α, IL–1β, and IL-18 became inactive. It can be concluded that ST has a certain inhibitory effect on the activation of the NLRP3 inflammasome and the subsequent inflammation that develops.

Previous research found that activation of the NLRP3 inflammasome results in endothelial injury in hypertension, while ST inhibits the expression of NLRP3 and caspase-1, restraining the activity of the NLRP3 inflammasome. Endothelia-dependent vasodilation function, as the main criterion to evaluate the status of endothelial function, has been internationally recognized. At present, we have a variety of methods to detect the relaxation of the endothelia. Detection of *in vitro* thoracic aortic ring tension was used to compare the vasodilation function differences in the vascular ring after Ach and SNP stimulation. The results indicated that the decrease in endothelia-dependent relaxation was the main manifestation in hypertension, which ST reversed, and this proved that ST protects against endothelial injury caused by hypertension. In our study, it was found that after ST intervention, eNOS and NO levels increased, ET-1 levels decreased, and the relative balance between vasoconstriction and relaxation was restored, which indicated that vascular endothelial function was improved due to the administration of ST.

During the process of hypertension, the vascular endothelia of the SHRs was damaged and shed to different degrees, the structure of the intima was discontinuous, the smooth muscle cells in the middle membrane were significantly proliferated, the fibers were thickened, bent, and fused, inflammatory cell infiltration was observed, and the vascular wall was significantly thickened. After ST intervention, the vascular endothelial structure was restored to intactness, the smooth muscle cells in the vascular wall did not significantly proliferate, and there was decreased inflammatory damage with increased vascular remodeling.

The regulation of blood pressure includes neuroregulation (mainly the sympathetic nervous system) (Daniels et al., 2017) and humoral regulation (mainly the RAAS) (Blanchard et al., 2006), while the NLRP3 inflammasome plays an important role in the neurohumoral regulation of hypertension. In pathological conditions, especially when cardiovascular risk factors are present, AngII is one of the important substances in the RAAS. As a strong vasoconstrictor, it induces vascular endothelial injury and endothelial dysfunction, and also gives rise to further inflammation of the cardiovascular system (Endtmann et al., 2011). Therefore, inhibition of NLRP3 inflammasome-mediated vascular endothelial injury is of great significance for the control of blood pressure and the protection of target organs. Because AngII is indispensable in endothelial cell injury in patients with hypertension (Arnold et al., 2013), we administered AngII to HUVECs, and then studied the protective effect of ST on endothelial cell function. We found that ST increased the proliferation, migration, and adhesion ability of HUVECs, promoted the formation of a tubular structure, and reduced AngII-induced endothelial dysfunction.

Endothelial dysfunction is defined as vasodilation dysfunction caused by an imbalance between vasoconstriction and vasodilation, which continuously contributes to the increase in blood pressure (Zahid et al., 2019). Our results showed that ST restored the balance between them, with increased eNOS and NO levels and decreased ET-1 level, indicating that the endothelial protection of ST was related to the promotion of the balance between vasoactive substances. The NLRP3 inflammasome can affect the production of NO, leading to endothelial dysfunction (Wang et al., 2018b). Our study found that the expression of inflammatory factors such as IL-1β and IL-18 produced and released by AngII-induced HUVECs increased, and the expression of NLRP3 and caspase-1 was also elevated. Studies have shown that IL-18 can directly promote the proliferation of vascular smooth muscle cells, eventually leading to an increase in blood pressure. After the addition of ST, a series of huvec inflammatory indicators were reduced, and the activation of the NLRP3 inflammasome was also significantly inhibited. Our experiments have shown that ST improves vascular endothelial function by inhibiting the activation of the NLRP3 inflammasome, thus protecting against the endothelial injury of hypertension.

The pathophysiological mechanism of hypertension is complex, and we focused on the endothelial injury associated with hypertension. Studies have found that the activation of the NLRP3 inflammasome can aggravate the vascular endothelial dysfunction, and ST can ameliorate the endothelial injury caused by hypertension by inhibiting the activation of the NLRP3 inflammasome, with a satisfactory antihypertensive effect. However, the antihypertensive mechanism of ST is intricate, and the current study considered the NLRP3 inflammasome only as an entry point for study. Only through subsequent multi-target and multi-pathway research on hypertensive endothelial injury can the key to the antihypertensive effect of ST be found, which can then effectively guide clinical practice and be applied as a treatment for hypertension as early as possible.

## Data Availability

The original contributions presented in the study are included in the article/Supplementary Material, further inquiries can be directed to the corresponding author.
