# Supplementary figures and images for: Sinapine Thiocyanate Ameliorates Vascular Endothelial Dysfunction in Hypertension by Inhibiting Activation of the NLRP3 Inflammasome
Source: Front Pharmacol. 2021 Feb 9;11:620159. doi: 10.3389/fphar.2020.620159 (PMC7901921; doi:10.3389/fphar.2020.620159)

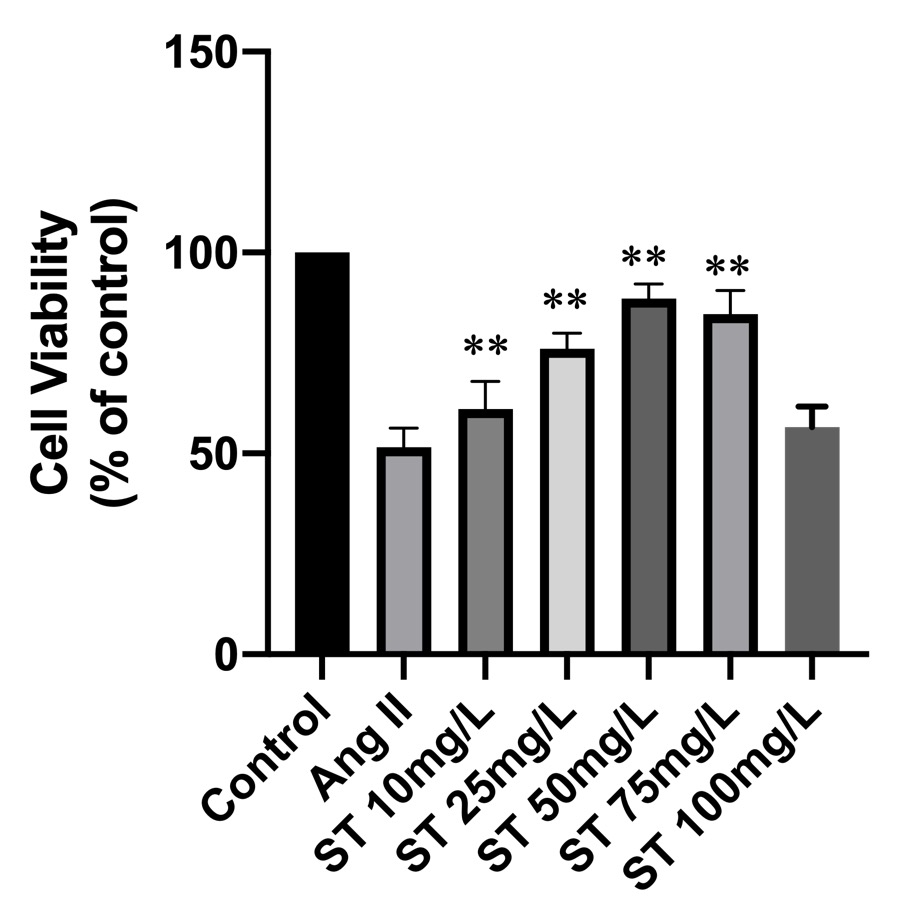

Supplement: Supplementary file 1 [file image1.jpeg]

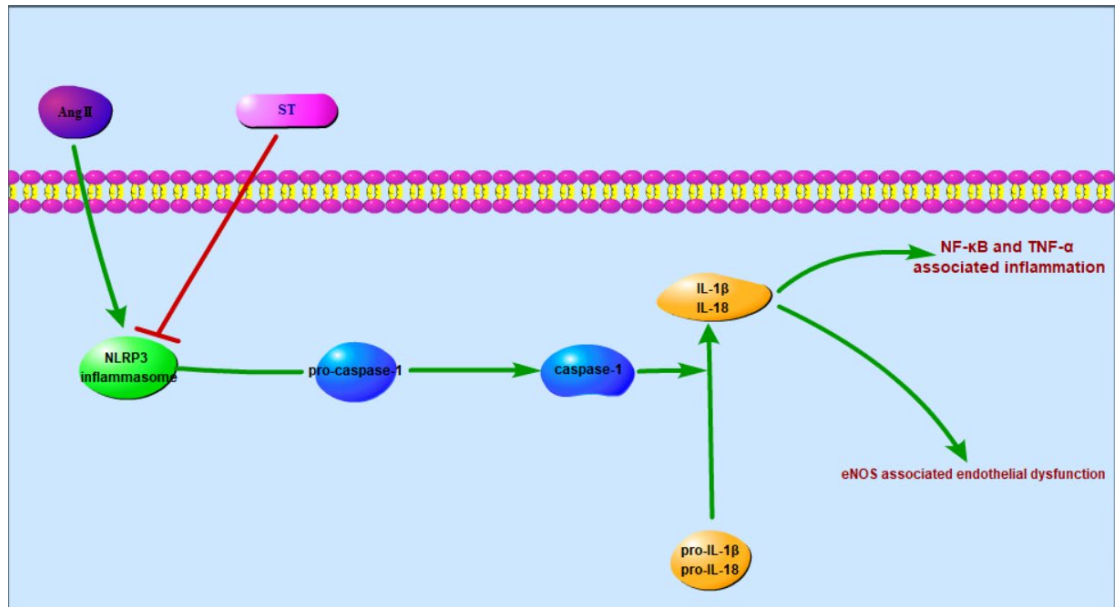

Supplement: Supplementary file 2 [file image2.pdf]
